# Supplementary figures and images for: Effects of Pieris japonica (Ericaceae) dominance on cool temperate forest altered-understory environments and soil microbiomes in Southern Japan
Source: PLoS One. 2024 Jan 11;19(1):e0296692. doi: 10.1371/journal.pone.0296692 (PMC10783712; doi:10.1371/journal.pone.0296692)

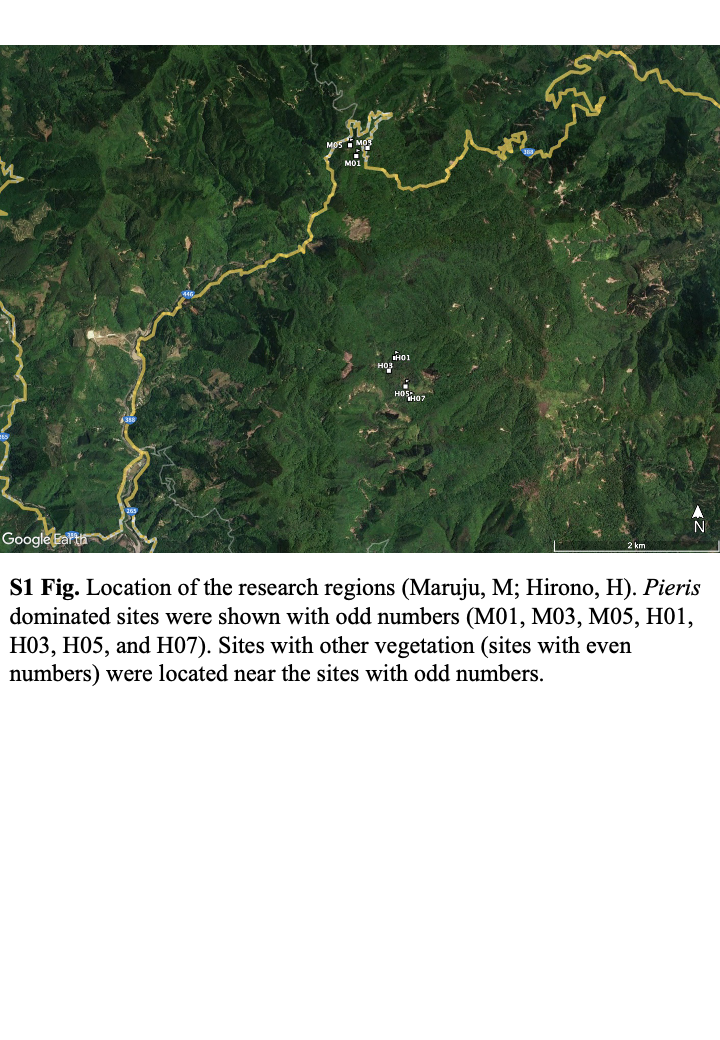

Supplement: S1 Fig — Pieris dominated sites were shown with odd numbers (M01, M03, M05, H01, H03, H05, and H07). Sites with other vegetation (sites with even numbers) were located near the sites with odd numbers. (TIFF) [file pone.0296692.s001.tiff]

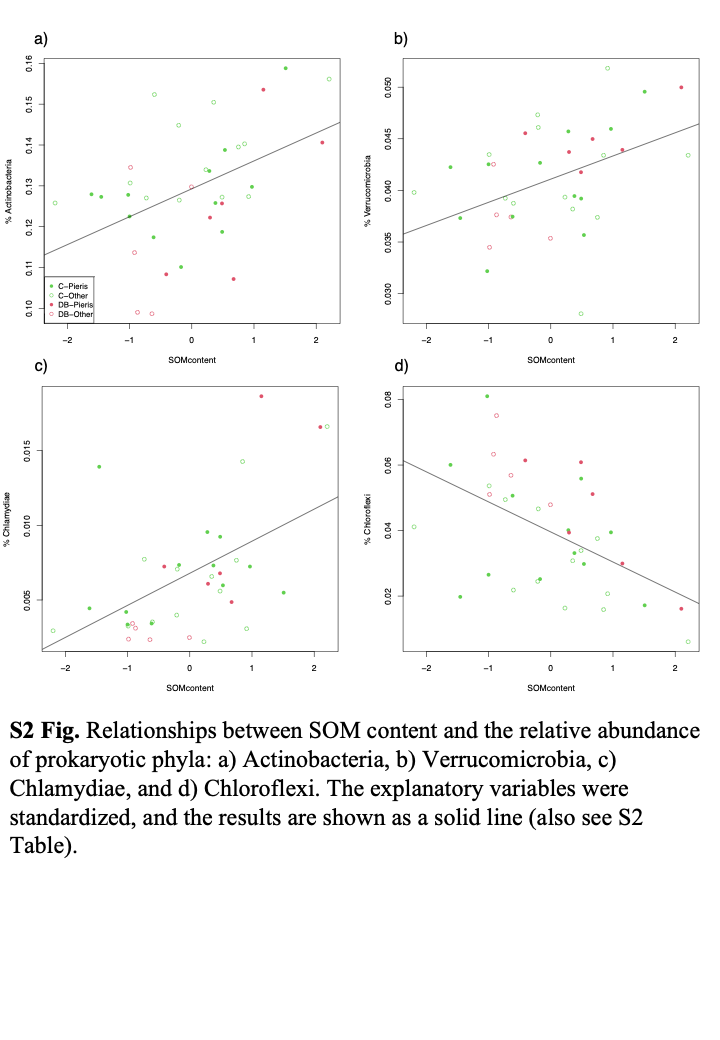

Supplement: S2 Fig — Relationships between SOM content and the relative abundance of prokaryotic phyla: a) Actinobacteria, b) Verrucomicrobia, c) Chlamydiae, and d) Chloroflexi. The explanatory variables were standardized, and the results are shown as a solid line (also see S2 Table). (TIFF) [file pone.0296692.s002.tiff]

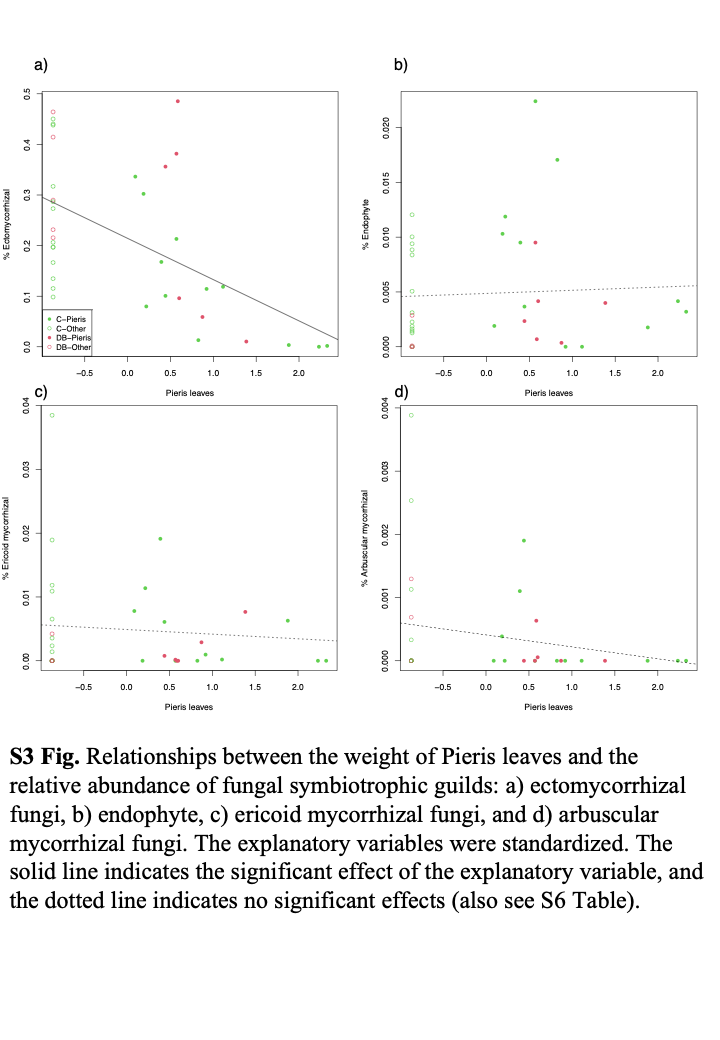

Supplement: S3 Fig — Relationships between the weight of Pieris leaves and the relative abundance of fungal symbiotrophic guilds: a) ectomycorrhizal fungi, b) endophyte, c) ericoid mycorrhizal fungi, and d) arbuscular mycorrhizal fungi. The explanatory variables were standardized. The solid line indicates the significant effect of the explanatory variable, and the dotted line indicates no significant effects (also see S6 Table). (TIFF) [file pone.0296692.s003.tiff]

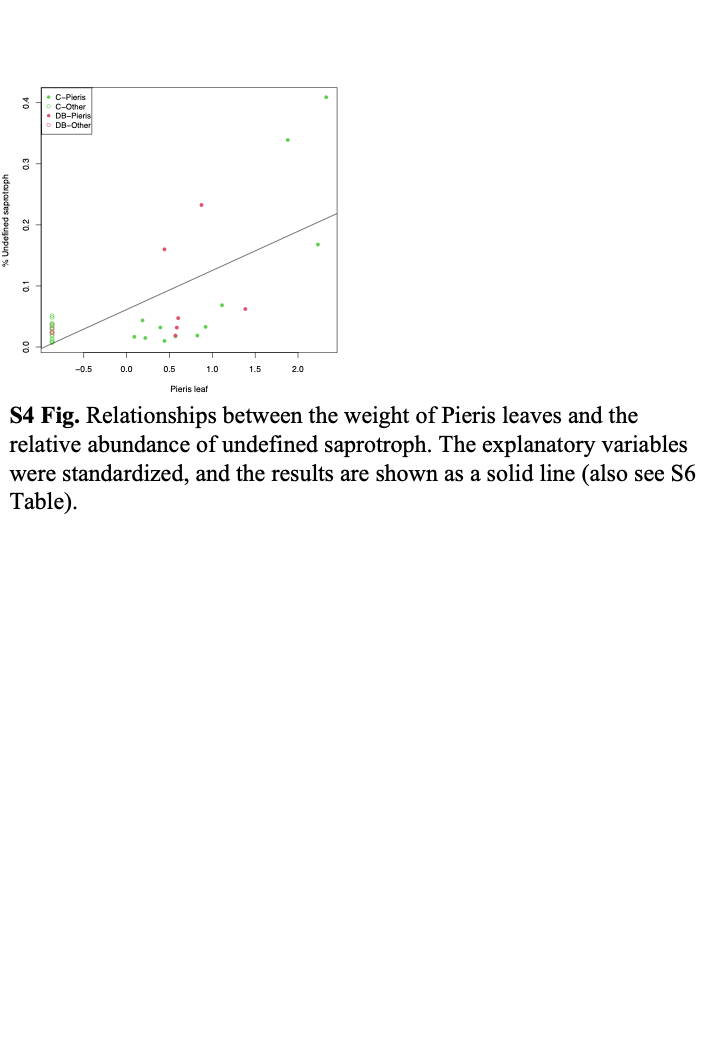

Supplement: S4 Fig — The explanatory variables were standardized, and the results are shown as a solid line (also see S6 Table). (TIFF) [file pone.0296692.s004.tiff]
