# Supplementary material for: Effects of Pieris japonica (Ericaceae) dominance on cool temperate forest altered-understory environments and soil microbiomes in Southern Japan
Source: PLoS One. 2024 Jan 11;19(1):e0296692. doi: 10.1371/journal.pone.0296692 (PMC10783712; doi:10.1371/journal.pone.0296692)
Supplement: Attachment — Submitted filename: Response to Reviewers.docx [file pone.0296692.s014.docx]

Response to the academic editor and reviewers’ comments on Ms. No. PONE-S-23-32909 Effects of Pieris japonica (Ericaceae) dominance on cool temperate forest altered-understory environments and soil microbiomes in Southern Japan

Response to the academic editor’s comments

Dear Professor, Dr. Jian Liu, Academic Editor of PLOS ONE,

Thank you very much for your review of our manuscripts.

We would like to reply to your comments below.

Journal Requirements:

Please review your reference list to ensure that it is complete and correct. If you have cited papers that have been retracted, please include the rationale for doing so in the manuscript text, or remove these references and replace them with relevant current references. Any changes to the reference list should be mentioned in the rebuttal letter that accompanies your revised manuscript. If you need to cite a retracted article, indicate the article’s retracted status in the References list and also include a citation and full reference for the retraction notice.

[Res] We reviewed the reference list to complete the correction. We changed the list as below.

- We enclosed the Issue number of Malik AU 2003 in parentheses.
- We made the mistake of citing two articles: Chamber et al. 2008 [9] and Walker et al. 1999 [46] while writing. We would like to replace Chamber et al. with Walker et al. and vice versa. We apologize for the mistake.
- The same reference was cited twice (12 and 29). After deleting 29, we corrected the number of citations after 29.
- The articles from 38 to 40 were changed in the order they appeared.
- Katayama et al. [41] have been published in the journal and we added issue and page numbers.

Additional Editor Comments:

The revised version has been improved a lot. But the manuscript still has some problems as suggested by the reviewer. The authors should respond to the comments of the reviewers one by one and revise the manuscript accordingly.

[Res] Thank you very much for your comments. We amended the manuscript files based in the reviewer’s comments. We would like you to confirm the documents again and would be grateful if the revised manuscript would meet the standard for the publication of PLOS ONE.

Sincerely,

Yuji Tokumoto, on behalf of the authors

Response to the reviewers’ comments

Dear Reviewer #2:

Thank you very much for the review and positive and constructive comments again. We would like to reply to the comments below.

Reviewer #2: In this version, the authors answered most questions from the editor and two reviewers and improved the quality of manuscript. There are only some minor questions need to be correct before publication.

In Environmental properties, if an index is significant between different sites, it is suggested to declare in which site it is higher or lower.

[Res] We modified the sentences that mentioned total litter weight, humus and Pieris leaf weights, soil bulk density and SOM content (second paragraph of section Environmental properties in Results).

Line 545-547, A previous study has compared the extracellular enzyme activities between sites with or without Ericaceae removal, and the presence of Ericaceae affects the microbial activities and soil C and N cycles [56]. Invalid citation. What is the main findings of this study? Did the presence of Ericaceae increase or decrease microbial activities, soil C and N cycles? Please check other citations, especially in discussion.

[Res] We modified the sentences that mentioned [55] (the previous number of the reference is 56) (L549-552). We checked the other citations throughout the text and found similar descriptions in (L55-57 and L523-524), so we also modified those sentences to introduce the findings of previous studies precisely.

Line 252-254, the reason for canopy openness change should be presented in discussion, not in results.

[Res] We moved the reason for canopy openness change to the first paragraph of the discussion. We have discussed the unfluctuating light conditions under the Pieris populations in L405-410.

There are some minor errors in format.

Table 2, the decimal of all data should be 0.01.

[Res] We amended it accordingly.

Line 150, Table 2, Line 235, Line 264, Line 312, Line 316, Line 319, Line 320, Line 321, Line 334, Line 347, Line 531, Line 535, Line 543, SOMcontent should be SOM content, a space symbol was missing.

[Res] We amended it accordingly.

Table 3, the decimal of all data should be 0.001.

[Res] We amended it accordingly.

Table 2, Table 3, Table 4, P-values should be p-values.

[Res] We amended it accordingly.

The first subtitle of discussion should be more concise. For example, Differences between understory environments according to Pieris dominance for different canopy trees.

[Res] We modified the subtitle to be concise as you kindly suggested.

Line 471, Pieris, the total word should be italic.

[Res] We amended it accordingly.

Line 588, 13 should not be bold.

[Res] We amended it accordingly.

Line 600, Line 606, Line 611, the article title should not be ended with a comma “,”.

[Res] We amended it accordingly. We found the same mistake in [3] and amended it.

Line 713, Line 745, last full stop “.” should be deleted.

[Res] We amended it accordingly.

Thank you for your review in details.

We hope our responses could meet your criterion for evaluation and revision.

Sincerely,

Yuji Tokumoto
